# The Effects of Intermittent Fasting on Inflammatory Markers in Adults: A Systematic Review and Pairwise and Network Meta-Analyses

**DOI:** 10.3390/nu17152388

**Published:** 2025-07-22

**Authors:** Mousa Khalafi, Aref Habibi Maleki, Shima Mojtahedi, Mahsa Ehsanifar, Sara K. Rosenkranz, Michael E. Symonds, Mohammad Sadegh Tarashi, Saeid Fatolahi, Maria Luz Fernandez

**Affiliations:** 1 Department of Sport Sciences, Faculty of Humanities, University of Kashan, Kashan 87317-53153, Iran; 2 Physiology Research Center, Iran University of Medical Sciences, Tehran 14496-14535, Iran; habibimaleki.a@iums.ac.ir; 3 Department of Exercise Physiology, Faculty of Physical Education and Sport Sciences, University of Tehran, Tehran 1417935840, Iran; shmojtahedi@ut.ac.ir (S.M.); ms.tarashi@ut.ac.ir (M.S.T.); 4 Department of Exercise Physiology and Corrective Exercises, Faculty of Sport Sciences, Urmia University, Urmia 5756151818, Iran; m.ehsanifar@urmia.ac.ir; 5 Department of Kinesiology and Nutrition Sciences, University of Nevada Las Vegas, Las Vegas, NV 89154, USA; sara.rosenkranz@unlv.edu; 6 Centre for Perinatal Research, Academic Unit of Population and Lifespan Sciences, School of Medicine, University of Nottingham, Nottingham NG7 2QL, UK; michael.symonds@nottingham.ac.uk; 7 Department of Physical Education and Sport Sciences, Faculty of Humanities, Tarbiat Modares University, Tehran 111-14115, Iran; saeed.ft1370@gmail.com; 8 Department of Nutritional Sciences, University of Connecticut, Storrs, CT 06269, USA

**Keywords:** intermittent fasting, adipokine, cytokine, CRP

## Abstract

**Background:** Intermittent fasting (IF) can improve inflammatory status, but its effects may be dependent on the mode of fasting. **Objectives:** We performed a systematic review with pairwise and network meta-analyses to investigate the effects of different modes of IF on inflammatory markers in adults. **Methods:** Three database searches were conducted, including PubMed, Scopus, and Web of Science, from inception to June 2024. The searches used two keyword groups: “intermittent fasting” and “inflammatory markers”. Randomized and non-randomized trials investigating any IF mode on inflammatory markers, including interleukin (IL)-6, tumor necrosis factor (TNF)α, C-reactive protein (CRP), leptin, and adiponectin, were included. Standardized mean differences (SMDs) were calculated using random effects models for both analyses. **Results:** A total of 21 studies (839 participants) were included. Compared with controls, IF reduced TNF-α [SMD: −0.31, *p* = 0.009], CRP [SMD: −0.19, *p* = 0.04], and leptin [SMD: −0.57, *p* = 0.005] but did not significantly affect IL-6 or adiponectin. Among the IF modes, time-restricted feeding (TRF) showed the largest reduction in TNF-α [−0.39, *p* = 0.001]. TRF had the highest probability ranking for changes in IL-6, TNF-α, leptin, and adiponectin; however, the effects on IL-6 and adiponectin were not statistically significant. The 5:2 diet ranked highest for CRP. **Conclusions:** IF may be an effective dietary therapy for improving some inflammatory markers, with effects potentially influenced by the mode of IF. TRF had the highest rankings across multiple markers, though the findings were not uniformly significant. Additional longer-term trials are needed to fully elucidate the anti-inflammatory potential of IF.

## 1. Introduction

Obesity has been associated with a number of metabolic diseases and, globally, has become one of the most important health challenges we face [1,2]. Mechanistically, obesity leads to structural and functional changes in adipocytes [3]. As they become enlarged, they lead to cellular changes, including the recruitment of immune cells, overproduction of pro-inflammatory markers, and enhanced secretion of adipokines [4,5]. Inflammatory markers, including interleukin-6 (IL-6), tumor necrosis-α (TNF-α), C-reactive protein (CRP), and adipokines, such as leptin and adiponectin, play central roles in obesity-induced inflammation [3,6,7], with weight loss ameliorating these effects and decreasing obesity-related adverse health outcomes [8,9,10].

Intermittent fasting (IF) is currently a popular approach for reducing body weight in populations with overweight or obesity [11,12,13]. Clinical and pre-clinical studies suggest that IF can be effective in improving cardiometabolic health markers, including blood pressure, lipid profiles, insulin resistance, and inflammation [12,13,14,15,16,17,18,19]. Previous meta-analyses examining the anti-inflammatory role of IF have yielded mixed results on inflammatory markers [3,7,20,21], with the mode of IF being an important moderator. The most prevalent types of IF include alternate-day fasting (ADF; fasting with 0–500 kcal intake every other day), time-restricted feeding (TRF; daily fasting periods longer than 12 h), and the 5:2 diet (two fasting days and five feeding days per week) [22,23]. Previous network meta-analyses have determined the most effective mode of IF for improving body composition, metabolic outcomes, and weight loss [24,25,26], but a similar approach has not been undertaken to identify which IF mode is most effective for reducing inflammation. Therefore, the aim of the current pairwise and network meta-analyses was to determine the effects of different modes of IF on the most commonly studied inflammatory markers in adults.

## 2. Materials and Methods

The current systematic review and meta-analysis was conducted according to the Preferred Reporting Items for Systematic Reviews and Meta-Analyses (PRISMA) checklist and the Cochrane Handbook for Systematic Reviews of Interventions. This systematic review was registered in the International Prospective Register of Systematic Reviews (PROSPERO) with ID: CRD42025644768.

### 2.1. Search Strategy

A comprehensive search was conducted in the three main databases, including PubMed, Scopus, and Web of Science, from inception to June 2024 using two keywords groups: (“time-restricted feeding” OR “time restricted feeding” OR “time-restricted eating” OR “time restricted eating” OR “time-restricted diet” OR “time restricted diet” OR “time-restricted fasting” OR “time restricted fasting” OR “intermittent fasting” OR “intermittent energy restriction” OR “alternate fasting” OR “periodic fasting” OR “reduced meal frequency” OR “alternate-day fasting” OR “alternate day fasting”) AND (“Inflammation” OR “inflammatory” OR “cytokine” OR “adipokine” OR “adipocytokine” OR “interleukin” OR “interleukin-6” OR “interleukin 6” OR “IL-6” OR “IL6” OR “interleukin-10” OR “interleukin 10” OR “IL-10” OR “IL 10” OR “tumor necrosis factor alpha” OR “TNF-α” OR “TNF α” OR “TNF” OR “C-Reactive protein” OR “Reactive protein” OR “hsCRP” OR “CRP” OR “leptin” or “adiponectin”). When available in the databases, the searches were limited to the English language and human participants. In addition, the reference lists of the included studies, Google Scholar, and previous meta-analyses [3,7,21] were manually searched. Details of the search strategy are available in Supplementary Table S1.

### 2.2. Study Selection and Inclusion and Exclusion Criteria

The results from the searches were exported into EndNote (version 21) by one author (M.K.). After removing duplicate records, study selection was conducted in two phases. First, the remaining studies were screened based on the titles/abstracts, and then the eligible studies were screened based on their full texts against the inclusion and exclusion criteria. Two authors (M.S.T., M.E.) then independently performed the study selection process, and any disagreements were resolved via discussion with another author (M.K.). The current meta-analysis was limited to randomized trials, except where high-quality non-randomized trials met all other eligibility criteria, in order to strengthen the analysis. We included journal articles written in English and peer-reviewed studies that met our eligibility criteria using the population, intervention, comparator, outcomes, and study design (PICOS) framework.

For the population, studies involving human participants aged ≥ 18 years, regardless of biological sex, who were either healthy or had chronic diseases, were included. Studies were included as follows: for interventions, studies testing any mode of IF, such as ADF, TRF, the 5:2 diet, or the 4:3 diet, with intervention durations ≥ 2 weeks; for comparators, studies with a non-intervention control group; and for outcomes, studies measuring circulating (serum or plasma) levels of inflammatory markers, including IL-6, TNFα, CRP, leptin, or adiponectin. The exclusion criteria were non-original studies, duplicate publications, and animal studies. In addition, studies on Ramadan fasting were excluded.

### 2.3. Data Extraction

Data from all included studies were extracted independently by two authors (M.S.T. and M.E.) and thoroughly reviewed by another author (A.H.M.). We extracted the following information and data: study characteristics, including first author name, publication year, sample size, and study design; participant characteristics, including health status, biological sex, age, and body mass index (BMI); intervention characteristics, including intervention duration, IF mode, and protocol; and inflammatory markers analyzed. To perform the meta-analysis, mean changes (post values–pre values) and their standard deviations (SDs) and sample sizes were extracted. However, when required, these data were calculated from baseline and post-intervention values using the relevant formula as recommended by the Cochrane Handbook. Data that were reported as standard errors, medians and interquartile ranges, and confidence intervals, were transformed to means and SDs [27,28,29]. For studies with multiple IF modes, each mode was included as a separate arm, and the sample size of the control group was divided by the number of intervention arms. If studies had missing data that were not extractable, the corresponding authors were contacted.

### 2.4. Quality Assessment

The overall quality of the included studies was assessed using the Physiotherapy Evidence Database (PEDro) scale, which is reliable for randomized trials. This tool contains 11 items, which are provided in the Supplementary Table S2. Each item was scored as “yes”, “no”, or “unclear”, with higher scores indicating higher study quality. The overall quality of the included studies was assessed by two authors (M.S.T. and M.E.) and thoroughly reviewed by another author (A.H.M.).

### 2.5. Statistical Analysis

To investigate the effects of IF versus controls, a pairwise meta-analysis was conducted for each outcome. Effect sizes (standardized mean differences) with corresponding 95% confidence intervals (95% CIs) were calculated using random effects models. These models were selected because of the assumption that heterogeneity was likely in clinical trials [30]. To assess heterogeneity, I^2^ statistics were calculated and interpreted as follows: I^2^ values < 30% indicated low heterogeneity, 30–70% indicated moderate heterogeneity, and >70% indicated high heterogeneity. Publication bias was assessed using visual interpretation of funnel plots and Egger’s tests with the significance level of *p* > 0.10. To investigate the effects of IF modes, including ADF, TRF, and the 5:2 diet, a network meta-analysis was conducted using the netmeta package in the statistical software R (V.4.4.1). Based on pre-established study heterogeneity, direct and indirect effect sizes were calculated using random-effects models with a frequentist framework. Similar to the pairwise meta-analysis, effect sizes (standardized mean differences) with corresponding 95% confidence intervals (95% CIs) were calculated for network meta-analysis. The network geometry was generated to display any relationships between intervention arms, forest plots were created to display the network estimations, and P-scores were calculated to rank the interventions according to probable effects. To assess heterogeneity, I^2^ statistics were used. To evaluate consistency, Q statistics were used to assess the within and total statistical heterogeneity. In addition, we used Egger’s tests and visual interpretation of funnel plots to detect publication bias.

## 3. Results

### 3.1. Study Characteristics

**Figure 1:**
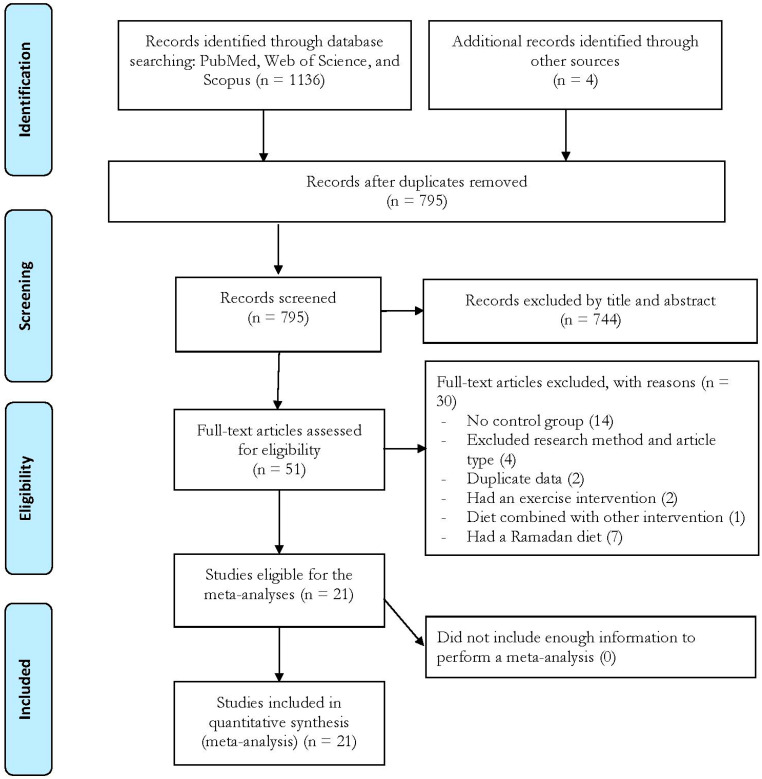
Flow diagram of the systematic literature search.

The online searches yielded 1136 records, and another 4 studies were included from manual searches. After removing duplicate records and preliminary screening, 51 studies remained for the full-text screening. Following the full-text screening, 21 studies [31,32,33,34,35,36,37,38,39,40,41,42,43,44,45,46,47,48,49,50,51,52] met all the inclusion criteria and were therefore included in the meta-analyses. The flow chart of this process is provided in Figure 1.

Among the included studies, 19 were randomized trials and 2 were non-randomized controlled trials [40,47]. In addition, 19 studies had parallel group study designs and 2 had crossover designs [42,49]. Data from one study were extracted from two different articles [31,32]. A total of 839 participants, ranging in age between 19 and 56 years and BMIs from 22 to 43 kg·m^2^, were included. The health status of the participants ranged from healthy to having chronic diseases, such as obesity and non-alcoholic fatty liver disease (NAFLD). The intervention durations ranged from 6 weeks to 12 months, lasting less than 12 weeks for the majority. Two modes of IF, namely, TRF and ADF, were used the most frequently, and one used the 5:2 diet [37] (Table 1). The quality scores of the included studies ranged from 4 (moderate quality) to 8 (high quality) (Supplementary Table S2).

### 3.2. Meta-Analyses

#### 3.2.1. IL-6

**Figure 2:**
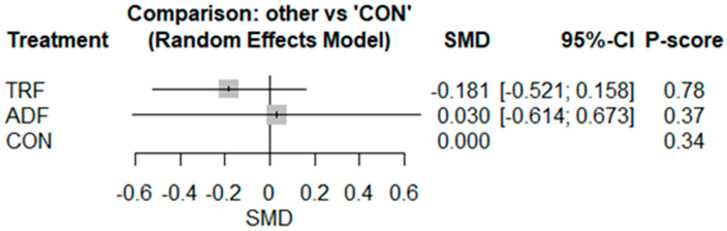
Forest plot of the network meta-analyses on IL-6. Data are reported as SMD (95% confidence limits). SMD: standardized mean difference.

The pairwise analysis indicated that IF had no significant effect on IL-6 [SMD: −0.13 (95% CI: −0.41 to 0.15), *p* = 0.37; I^2^ = 35.09, *p* = 0.12] compared to CON (Supplementary Figure S1). There was no publication bias indicated by visual interpretation of the funnel plot and Egger’s test (*p* = 0.14). The network geometry and meta-analysis included results from 10 studies involving 10 pairwise comparisons, 3 treatment arms, and 2 study designs (Supplementary Figure S2). The results showed no significant effects of TRF [−0.18 (95% CI −0.52 to 0.15), *p* = 0.29] or ADF [0.02 (95% CI −0.61 to 0.67), *p* = 0.92] as compared with CON (Figure 2). Based on the P-score-based rankings, the highest ranking was for TRF (0.78), followed by ADF (0.37), and the control (0.34) (Figure 2).

The results of the heterogeneity and inconsistency tests demonstrated moderate heterogeneity, with I^2^ = 40.1% [0.0%; 72.4%], and non-significant inconsistency (total designs), with Q = 13.35, df = 8, and *p* = 0.10. The Egger’s test did not show significant publication bias (*p* = 0.15) (Supplementary Figure S3).

#### 3.2.2. TNF-α

**Figure 3:**
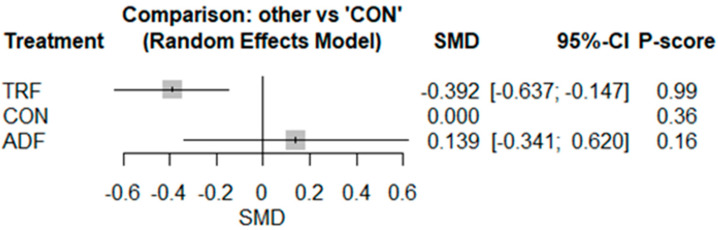
Forest plot of the network meta-analyses on TNF-α. Data are reported as SMD (95% confidence limits). SMD: standardized mean difference.

The pairwise analysis illustrated that IF decreased TNF-α [SMD: −0.31 (95% CI: −0.55 to −0.07), *p* = 0.009; I^2^ = 11.92, *p* = 0.33] significantly more than CON (Supplementary Figure S4), with no publication bias based on visual interpretation of the funnel plot and Egger’s test (*p* = 0.90). The network geometry and meta-analysis for TNF-α included results from nine studies involving nine pairwise comparisons, three treatment arms, and two study designs (Supplementary Figure S5). The results indicated that TRF decreased TNF-α [−0.39 (95% CI −0.63 to −0.14), *p* = 0.001] significantly more than CON, but there were no significant effects of ADF as compared with CON [0.13 (95% CI −0.34 to 0.61), *p* = 0.57] (Figure 3).

Based on the P-score-based rankings, the highest ranking was for TRF (0.99), followed by CON (0.36) and ADF (0.16) (Figure 3). The results of the heterogeneity and inconsistency tests demonstrated no significant heterogeneity, with I^2^ = 0.0% [0.0%; 67.6%] (although the confidence interval was wide), and non-significant inconsistency (total designs), with Q = 5.75, df = 7, and *p* = 0.56. Visual interpretation of the funnel plot did not suggest publication bias (Supplementary Figure S6).

#### 3.2.3. CRP

The pairwise analysis demonstrated that IF decreased CRP [SMD: −0.19 (95% CI: −0.39 to −0.00), *p* = 0.04; I^2^ = 21.18, *p* = 0.22] significantly more than CON (Supplementary Figure S7). Visual interpretation of the funnel plot suggested publication bias, but bias was not confirmed by the Egger’s test results (*p* = 0.26). The network geometry and meta-analysis for CRP included results from 14 studies involving 14 pairwise comparisons, 4 treatment arms, and 3 study designs (Supplementary Figure S8). The results showed no significant differences between the 5:2 diet [−0.64 (95% CI −1.34 to 0.05), *p* = 0.07], ADF [−0.19 (95% CI −0.62 to 0.24), *p* = 0.38], or TRF [−0.14 (95% CI −0.37 to 0.09), *p* = 0.23].

**Figure 4:**
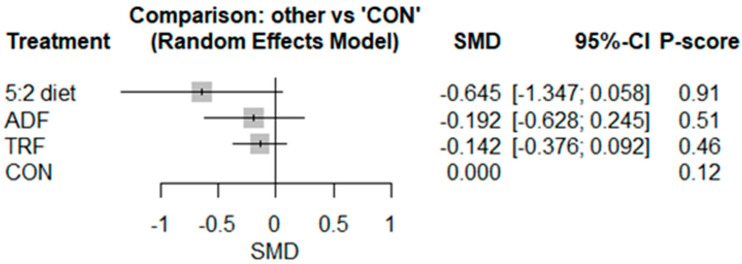
Forest plot of the network meta-analyses on CRP. Data are reported as SMD (95% confidence limits). SMD: standardized mean difference.

Based on the P-score-based rankings, the highest ranking was for the 5:2 diet (0.91), followed by ADF (0.51), TRF (0.46), and CON (0.12) (Figure 4).

The results of the heterogeneity and inconsistency tests demonstrated low heterogeneity, with I^2^ = 23.9% [0.0%; 61.2%], and non-significant inconsistency (total designs), with Q = 14.46, df = 11, and *p* = 0.20. The Egger’s test did not show significant publication bias (*p* = 0.57) (Supplementary Figure S9).

#### 3.2.4. Leptin

**Figure 5:**
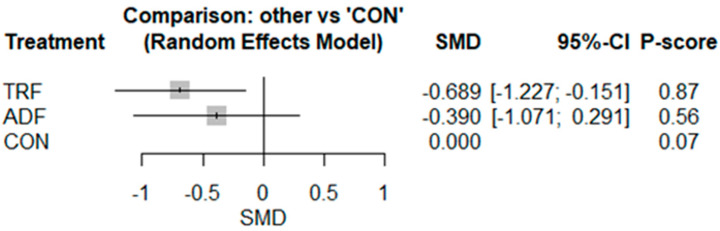
Forest plot of the network meta-analyses on leptin. Data are reported as SMD (95% confidence limits). SMD: standardized mean difference.

The pairwise analysis indicated that IF decreased leptin [SMD: −0.57 (95% CI: −0.97 to −0.17), *p* = 0.005; I^2^ = 63.12, *p* = 0.008] significantly more than CON (Supplementary Figure S10). Visual interpretation of the funnel plot suggested publication bias, but the Egger’s test (*p* = 0.49) did not confirm such bias. The network geometry and meta-analysis for leptin include results from eight studies involving eight pairwise comparisons, three treatment arms, and two study designs (Supplementary Figure S11). TRF showed the strongest reduction in leptin [−0.68 (95% CI −1.22 to −0.15), *p* = 0.01] compared to CON (Figure 5), although the effects varied between studies. No significant effect was observed for ADF [−0.39 (95% CI −1.07 to 0.29), *p* = 0.26] (Figure 5). Based on the P-score-based rankings, the highest ranking was for TRF (0.87), followed by ADF (0.56) and CON (0.07) (Figure 5). The results of the heterogeneity and inconsistency tests demonstrated moderate heterogeneity, with I^2^ = 66.4% [25.0%; 85.0%], and significant inconsistency (total designs), with Q = 17.86, df = 6, and *p* = 0.006. Visual interpretation of the funnel plot did not suggest publication bias (Supplementary Figure S12).

#### 3.2.5. Adiponectin

**Figure 6:**
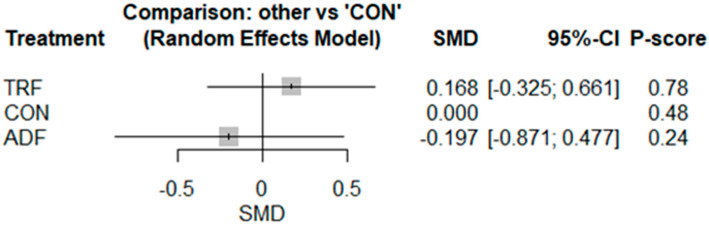
Forest plot of the network meta-analyses on adiponectin. Data are reported as SMD (95% confidence limits). SMD: standardized mean difference.

The pairwise analysis illustrated that IF did not increase adiponectin [SMD: 0.03 (95% CI: −0.34 to 0.41), *p* = 0.85; I^2^ = 61.48, *p* = 0.008] significantly more than CON (Supplementary Figure S13), and visual interpretation of the funnel plot did not suggest publication bias. However, publication bias was suggested by the Egger’s test results (*p* = 0.03). The network geometry and meta-analysis for adiponectin included results from nine studies involving nine pairwise comparisons, three treatment arms, and two study designs (Supplementary Figure S14). The results showed no significant differences between ADF [−0.19 (95% CI −0.87 to 0.47), *p* = 0.56] or TRF [0.16 (95% CI −0.32 to 0.66), *p* = 0.50] and the controls (Figure 6).

Based on the P-score-based rankings, the highest ranking was for TRF (0.78), followed by CON (0.48) and ADF (0.24) (Figure 6). The results of the heterogeneity and inconsistency tests demonstrated moderate heterogeneity, with I^2^ = 64.6% [24.5%; 83.4%], and significant inconsistency (total), with Q = 19.70, df = 7, and *p* = 0.006. Visual interpretation of the funnel plot did not suggest publication bias (Supplementary Figure S15).

## 4. Discussion

This systematic review and pairwise and network meta-analyses revealed that overall, IF was effective for reducing TNF-α, CRP, and leptin, as compared with control groups. The network meta-analyses indicated that the highest probability ranking order was as follows: TRF ranked highest for IL-6, TNF-α, leptin, and adiponectin, though the effects on IL-6 and adiponectin were not statistically significant, and the 5:2 diet for CRP. TRF had the highest P-score ranking for multiple pro-inflammatory markers as compared with the other modes of IF. IF is a popular dietary intervention that can be effective in improving body composition, insulin resistance, lipid profiles, blood pressure, and liver function, and it is therefore considered an alternative to continuous energy restriction [13,15,53]. To date, several systematic reviews have investigated the efficacy of IF in improving inflammation [3,7,21]. To elucidate the different anti-inflammatory effects of various IF modes, we conducted both pairwise and network meta-analyses to compare three of the most studied modes of IF, including TRF, ADF, and the 5:2 diet.

IL-6 is an important inflammatory cytokine that plays dual pro- and anti-inflammatory roles [54], depending on its source. In people with obesity and related comorbid conditions, IL-6 secreted from adipose tissue contributes to the production of hepatic CRP and a pro-inflammatory environment [55,56,57,58]. Assessing IL-6 can be important for informing both disease progression and treatment [59], with caloric restriction-induced weight loss inhibiting the secretion of IL-6 from adipose tissue [9]. Despite the role of IF in weight loss, our findings align with previous meta-analyses showing no significant effects of IF on IL-6 [SMD: −0.13 (95% CI: −0.41 to 0.15), *p* = 0.37] [3,7,21]. Several factors, including obesity, insulin resistance, metabolic disorders, intervention durations, and modes, affect inflammatory responses to different therapies. Therefore, heterogeneity in participants and intervention durations may be important moderators of the effects of IF on IL-6 [3]. The majority of the included studies had intervention durations lasting less than 12 weeks, suggesting that a longer-term intervention may be needed to reduce IL-6 as compared with CON.

A related inflammatory marker worth noting is TNF-α. TNF-α is the primary pro-inflammatory cytokine produced by macrophages and monocytes, contributing to the pathogenesis of autoimmune diseases [60] and acting as a metabolic messenger produced by adipose tissue, ultimately contributing to obesity-related metabolic diseases [61,62,63,64]. Therefore, inhibiting the production and secretion of TNF-α is important, and weight loss is an effective approach for reducing systemic levels [64,65]. IF is an effective weight loss strategy, and our findings confirm a reduction in TNF-α, which is in alignment with previous meta-analyses on people with overweight and obesity [7] and in the general adult population [3]. However, another meta-analysis did not show beneficial effects on TNF-α [21]. The variety in the modes of IF, the participant characteristics, the methodologies of the included studies, and the number and quality of the included studies may be important factors explaining these inconsistent findings. In addition, the IF mode may be an important moderator in the efficacy of specific IF interventions for reducing TNF-α. The current results showed that TRF was the only IF mode that was effective for reducing TNF-α, and it was the highest-ranked IF mode among those studied. The mechanisms underlying this effect remain unclear. Several network meta-analyses have reported that ADF was more effective for improving body composition and metabolic health when compared with other IF modes [24,26]. TRF, defined as limiting caloric intake to a daily time-restricted window, typically followed by fasting periods longer than 12 h [23], may be effective for reducing TNFα, not only due to reductions in weight and fat mass but also due to decreases in the quantity of macrophages within adipose tissue. However, whether these effects are independent of weight loss remains unclear. Given this, the subsequent evidence favoring ADF for improving body composition may also be relevant to inflammation-related outcomes. Some research also suggests the plausible realignment of circadian clocks during TRF, which may indirectly play a role in weight and fat mass reductions [3,66].

Another commonly studied marker is CRP, a marker of chronic low-grade inflammation and a predictor of cardiovascular and metabolic diseases. CRP is known to be elevated in obesity and metabolic diseases [55,67,68,69]. CRP can be effectively reduced with lifestyle interventions, including exercise training and dietary modifications [70,71,72,73]. Several meta-analyses have confirmed the anti-inflammatory effects of IF through reductions in CRP, but the results have been somewhat mixed [3,7,21]. Similar to findings on IL-6 and TNF-α, the small number of studies included in the meta-analyses and the variability in the participant characteristics and interventions may explain these mixed results. Decreases in CRP with 5:2 diets may be explained by loss in body weight and fat mass and improvements in pro-inflammatory cytokines, such as TNF-α, but this study only recruited patients with NAFLD [37].

In addition to CRP and TNF-α, leptin has also been studied in this context. Leptin is well known as one of the main suppressors of appetite, promoting satiety and signaling to the brain that there are sufficient energy stores available [74,75]. Leptin has a dual role as a hormone and a cytokine, contributing to the generation and maintenance of low-grade inflammation [76,77]. The current results showed that IF effectively reduced leptin, and TRF was the particular mode of IF that was more beneficial, as compared to the other modes. Similarly, one previous meta-analysis also showed that fasting and energy-restricted diets may reduce leptin [78]. Leptin production is proportional to body fat mass; therefore, weight loss and reducing fat mass, especially reducing visceral fat, may explain the beneficial effects of IF on reducing leptin because adipose tissue acts as an endocrine organ. Therefore, reductions in adipose tissue are expected to lower circulating leptin [79,80].

Adiponectin is often examined alongside leptin, and it is produced primarily in adipose tissue. Adiponectin has anti-obesity effects through its anti-hyperglycemic, anti-atherogenic, and anti-inflammatory properties [81]. Although adiponectin can be increased through weight loss [82], any effects of IF are unclear. The current results demonstrated that IF had no effect on adiponectin, supporting previous meta-analyses that show no effects of fasting and energy-restricted diets [78], Ramadan intermittent fasting [83], or TRF [3]. These findings indicate that increases in adiponectin may not be among the beneficial effects of IF, but this should be carefully interpreted considering the small number of studies and the short durations of the interventions.

Our study has several limitations. Although pairwise and network meta-analyses were conducted, the small number of available studies for some comparisons may affect the validity of the results. In addition, the majority of the included studies had relatively short-term durations that may not be sufficient to achieve beneficial effects. We included adults with various health conditions, including both healthy adults and those with chronic diseases. These different populations likely had different baseline levels of inflammatory status and thus different responses to IF. Finally, there were insufficient data to distinguish whether the observed effects were attributable to the fasting interventions themselves or were secondary to weight loss and changes in energy balance.

## 5. Conclusions

IF may be an effective intervention for reducing TNF-α, CRP, and leptin, which may contribute to beneficial cardiometabolic outcomes as well as other health-related outcomes. TRF ranked highest in the network analyses for IL-6, TNF-α, leptin, and adiponectin, although statistical significance was not consistently observed. The 5:2 diet ranked the highest for reducing CRP. Additional randomized trials with longer durations and larger sample sizes are warranted to better determine the effectiveness of various IF modes on inflammatory markers, as the current findings do not support definitive conclusions.

## Figures and Tables

**Table 1:** Characteristics of participants and interventions.

Source, Year	Participant Characteristics				Intervention Characteristics					
	Sample Size (Sex)	Health Status	Age (Years)	BMI (kg/m^2^)	Design	Duration (Weeks or Months)	IF Mode	IF Protocol	CON or CR Protocol	Outcomes
Bhutani et al. 2013 [31,32]	32 (F and M)	Obese	IF: 42.0 ± 2.0 CON: 49.0 ± 2.0	IF: 35.0 ± 1.0 CON: 35.0 ± 1.0	RCT	12 weeks	ADF	Consumed 25% of baseline energy on fasting days (24 h) and ate ad libitum on feeding days (24 h)	CON: Maintained normal diet	Adiponectin, Leptin, CRP
Cho et al. 2019 [33]	13 (F and M)	Overweight/ Obese	IF: 33.5 ± 5.0 CON: 42.6 ± 10.6	IF: 27.8 ± 3.4 CON: 25.8 ± 3.4	RCT	8 weeks	ADF	Consumed 25% of their daily recommended energy intake (approximately 500 kcal)	CON: Normal daily habits	CRP
Cienfuegos et al. 2020 [34]	49 (F and M)	Obese	IF1: 49.0 ± 2.0 IF2: 46.0 ± 3.0 CON: 45.0 ± 2.0	IF1: 36.0 ± 1.0 IF2: 37.0 ± 1.0 CON: 36.0 ± 1.0	RCT	8 weeks	TRF	IF1: Ate ad libitum from 3 to 7 p.m. daily (20 h fast) IF2: Ate ad libitum from 1 to 7 p.m. daily (18 h fast)	CON: No meal timing restrictions	TNF-α, IL-6
Gabel et al. 2019 [35]	43 (F and M)	Overweight/ Obese	IF: 43.1 ± 9.9 CR: 42.0 ± 12.4 CON: 41.0 ± 11.6	IF: 34.0 ± 3.3 CR: 36.0 ± 4.1 CON: 35.0 ± 3.9	RCT	12 months	ADF	Consumed 25% of their baseline energy needs at lunch (between 12 and 2 p.m.)	CR: Consumed 75% baseline energy CON: Did not change their usual eating and activity habits	hsCRP, TNF-α, IL-6
Haganes et al. 2022 [36]	66 (F)	Overweight/ Obese	IF: 36.2 ± 5.9 CON: 36.4 ± 6.2	IF: 31.8 ± 3.3 CON: 33.1 ± 4.2	RCT	7 weeks	TRF	A ≤10 h daily eating window with ad libitum energy intake	CON: Not to change their diets	Adiponectin, Leptin
Kord Varkaneh et al. 2022 [37]	44 (F and M)	NAFLD	IF: 46.4 ± 13.4 CON: 44.2 ± 4.9	IF: 30.4 ± 2.3 CON: 30.6 ± 3.1	RCT	12 weeks	5:2 diet	On fasting days, 25% of recommended calorie intake from 12 to 2 p.m.	CON: Maintenance of usual diet	hsCRP
Kord Varkaneh et al. 2023 [38]	45 (F and M)	NAFLD	IF: 41.4 ± 10.5 CON: 44.2 ± 4.9	IF: 29.1 ± 2.6 CON: 30.6 ± 3.1	RCT	12 weeks	TRF	Maintained 16 h fasting/8 h feeding daily plus a low-sugar diet	CON: Diet based on traditional meals	hsCRP
Kotarsky et al. 2021 [39]	21 (F and M)	Overweight/ Obese	IF: 45.0 ± 9.9 CON: 44.0 ± 6.3	IF: 29.8 ± 2.6 CON: 29.4 ± 2.5	RCT	8 weeks	TRF	Consumed all their calories between 12:00 p.m. and 8:00 p.m. each day, inducing a fasting window of 16 h	CON: Maintained their regular eating schedule	CRP
Lao et al. 2023 [40]	27 (F and M)	CKD	IF: 51.8 ± 7.7 CON: 52.5 ± 11.3	IF: 29.3 ± 2.3 CON: 28.0 ± 2.4	NRCT	12 weeks	TRF	Followed a low-protein diet, eating three meals within an 8 h window starting between 7:00 a.m. and noon. During fasting periods, only water and non-caloric beverages were allowed	CON: Received a high-quality low-protein diet with no restrictions on what time they could eat each day, following their daily routines	CRP, TNF-α, IL-6
Manoogian et al. 2022 [41]	137 (F and M)	Healthy	IF: 41.1 ± 8.7 CON: 39.6 ± 9.4	IF: 27.8 ± 3.6 CON: 27.7 ± 3.9	RCT	12 weeks	TRF	Followed a 14 h fast, 10 h eating window; self-selected: 09:00–19:00 (60% CHO, 25% fat, 15% protein); average 11.3 h eating window	CON: Standard care (Mediterranean diet)	CRP
Martens et al. 2020 [42]	22 (F and M)	Healthy	67.0 ± 1.0	24.7 ± 0.6	RXT	6 weeks	TRF	Maintained 16 h of daily fasting and eating during the other 8 h	CON: Standard care	CRP, IL-6
Miranda et al. 2018 [43]	42 (F and M)	Obese	IF: 44.0 ± 21.4 CON: 43.0 ± 24.8	IF: 33.0 ± 6.3 CON: 34.5 ± 7.2	RCT	12 weeks	ADF	Consumed 25% of baseline energy needs as a lunch (12 p.m.–2 p.m.) on “fast days” and 125% of their energy needs across three meals on subsequent “feast days”	CON: Not to change their diet	Adiponectin, Leptin, TNF-α, IL-6
Moro et al. 2016 [44]	34 (M)	Healthy	IF: 29.9 ± 4.1 CON: 28.5 ± 3.5	ND	RCT	8 weeks	TRF	Consumed 100% of daily energy needs within an 8 h time window (at 1 p.m., 4 p.m., and 8 p.m.)	CON: Caloric intake as three meals consumed at 8 a.m., 1 p.m., and 8 p.m.	Adiponectin, Leptin, TNF-α, IL-6
Moro et al. 2020 [45]	16 (M)	Healthy	IF: 19.4 ± 2.4 CON: 19.4 ± 1.6	IF: 21.9 ± 1.7 CON: 22.5 ± 1.8	RCT	4 weeks	TRF	Consumed 100% of estimated daily energy needs in an 8 h time window (from 10:00 a.m. to 6:00 p.m.)	CON: Consumed a complete diet divided into three meals between 7 a.m. and 9 p.m.	Adiponectin, TNF-α, IL-6
Moro et al. 2021 [46]	20 (M)	Healthy	ND	ND	RCT	12 months	TRF	Consumed 100% of daily energy needs within an 8 h time window (at 1 p.m., 4 p.m., and 8 p.m.)	CON: Caloric intake as three meals consumed at 8 a.m., 1 p.m., and 8 p.m.	Adiponectin, Leptin, TNF-α, IL-6
Schroder et al. 2021 [47]	22 (F)	Obese	IF: 36.6 ± 1.6 CON: 42.3 ± 3.5	IF: 32.5 ± 1.1 CON: 43.5 ± 1.2	NRCT	3 months	TRF	Fasting period (no energy intake whatsoever) of 16 h (8 p.m. to 12 p.m.) and ad libitum feeding for 8 h (12 p.m. to 8 p.m.)	CON: Maintained their habitual nutrition throughout the whole period	CRP
Stratton et al. 2020 [48]	26 (M)	Healthy	IF: 22.9 ± 3.6 CON: 22.5 ± 2.2	ND	RCT	4 weeks	TRF	Maintained 25% caloric deficit and only ate within an 8 h window each day	CON: 25% caloric deficit with participants’ usual daily feeding schedules	Adiponectin, Leptin
Sutton et al. 2018 [49]	8 (M)	Prediabetes	56.0 ± 9.0	32.2 ± 4.4	RXT	5 weeks	TRF	Maintained 6 h feeding period, with dinner before 3 p.m.	CON: 12 h feeding period	CRP, IL-6
Varady et al. 2013 [50]	30 (F and M)	Healthy	IF: 47.0 ± 11.6 CON: 48.0 ± 7.7	IF: 26.0 ± 3.9 CON: 26.0 ± 3.9	RCT	12 weeks	ADF	Consumed 25% of their baseline energy needs on the fast day and then ate ad libitum on each alternating feed day	CON: Ate ad libitum	Adiponectin, Leptin, CRP
Xie et al. 2022 [51]	82 (F and M)	Healthy	IF1: 28.7 ± 9.7 IF2: 31.1 ± 8.4 CON: 33.6 ± 11.6	IF1: 22.7 ± 3.1 IF2: 21.4 ± 2.2 CON: 21.5 ± 2.9	RCT	5 weeks	TRF	IF1: (16 h fasting:8 h feeding) self-selected feeding window between 06:00 and 15:00 IF2: (16 h fasting:8 h feeding) self-selected feeding window between 11:00 and 20:00	CON: Ate ad libitum	Leptin, TNF-α, CRP
Zhang et al. 2022 [52]	60 (F and M)	Overweight/ Obese	IF1: 23.8 ± 2.7 IF2: 23.2 ± 2.2 CON: 21.1 ± 1.7	IF1: 27.1 ± 3.2 IF2: 28.5 ± 3.6 CON: 27.8 ± 3.5	RCT	8 weeks	TRF	IF1: 6 h eating window from 7:00 to 13:00 IF2: 6 h eating window from 12:00 to 18:00	CON: Ate ad libitum	

Abbreviations: IF—intermittent fasting, CR—calorie restriction, CON—control, BMI—body mass index, F—female, M—male, NAFLD—non-alcoholic fatty liver disease, RCT—randomized control trial, TRF—time-restricted feeding, RXT—randomized crossover trial, NRCT—non-randomized control trial, CKD—chronic kidney disease, CRP—C-reactive protein, TNF-α—tumor necrosis factor-alpha, IL-6—interleukin-6.

## Data Availability

The original contributions presented in this study are included in this article/Supplementary Material. Further inquiries can be directed to the corresponding authors.

## Supplementary Materials

The following supporting information can be downloaded at: https://www.mdpi.com/article/10.3390/nu17152388/s1, Table S1. Search strategy; Table S2. Risk of bias assessment; Figure S1. Forest plot of the effects of IF training versus CON on IL-6; Figure S2. Network geometric map of studies investigating the effect of IF training on IL-6; Figure S3. Network meta-analysis of funnel plots for IL-6; Figure S4. Forest plot of the effects of IF training versus CON on TNF-α; Figure S5. Network geometric map of studies investigating the effect of IF training on TNF-α; Figure S6. Network meta-analysis of funnel plots for TNF-α; Figure S7. Forest plot of the effects of IF training versus CON on CRP; Figure S8. Network geometric map of studies investigating the effect of IF training on CRP; Figure S9. Network meta-analysis of funnel plots for CRP; Figure S10. Forest plot of the effects of IF training versus CON on leptin; Figure S11. Network geometric map of studies investigating the effect of IF training on leptin; Figure S12. Network meta-analysis of funnel plots for leptin; Figure S13. Forest plot of the effects of IF training versus CON on adiponectin; Figure S14. Network geometric map of studies investigating the effect of IF training on adiponectin; Figure S15. Network meta-analysis of funnel plots for adiponectin.

## Abbreviations

IF	Intermittent Fasting
IL-6	Interleukin-6
TNF-α	Tumor Necrosis Factor-Alpha
CRP	C-Reactive Protein
hsCRP	High-Sensitivity C-Reactive Protein
SMD	Standardized Mean Difference
TRF	Time-Restricted Feeding
ADF	Alternate-Day Fasting
NAFLD	Non-Alcoholic Fatty Liver Disease
BMI	Body Mass Index
