# Supplementary material for: The Effects of Intermittent Fasting on Inflammatory Markers in Adults: A Systematic Review and Pairwise and Network Meta-Analyses
Source: Nutrients. 2025 Jul 22;17(15):2388. doi: 10.3390/nu17152388 (PMC12348594; doi:10.3390/nu17152388)
Supplement: Supplementary file 1 [file nutrients-17-02388-s001.zip › nutrients-3748508-supplementary.pdf]

Review

The Effects of Intermittent Fasting on Inflammatory Markers in Adults: A Systematic Review and Pairwise and Network Meta-Analyses

Mousa Khalafi [1,*], Aref Habibi Maleki [2], Shima Mojtahedi [3], Mahsa Ehsanifar [4], Sara K. Rosenkranz [5], Michael E. Symonds [6], Mohammad Sadegh Tarashi [3], Saeid Fatolahi [7] and Maria-Luz Fernandez [8,*]

1 Department of Sport Sciences, Faculty of Humanities, University of Kashan, Kashan 87317-53153, Iran
2 Physiology Research Center, Iran University of Medical Sciences, Tehran 14496-14535, Iran; habibimaleki.a@iums.ac.ir
3 Department of Exercise Physiology, Faculty of Physical Education and Sport Sciences, University of Tehran, Tehran 1417935840, Iran; shmojtahedi@ut.ac.ir (S.M.); ms.tarashi@ut.ac.ir (M.S.T.)
4 Department of Exercise Physiology and Corrective Exercises, Faculty of Sport Sciences, Urmia University, Urmia 5756151818, Iran; m.ehsanifar@urmia.ac.ir
5 Department of Kinesiology and Nutrition Sciences, University of Nevada Las Vegas, Las Vegas, NV 89154, USA; sara.rosenkranz@unlv.edu,
6 Centre for Perinatal Research, Academic Unit of Population and Lifespan Sciences, School of Medicine, University of Nottingham, Nottingham NG7 2QL, UK; michael.symonds@nottingham.ac.uk
7 Department of Physical Education and Sport Sciences, Faculty of Humanities, Tarbiat Modares University, Tehran 111-14115, Iran; saeed.ft1370@gmail.com
8 Department of Nutritional Sciences, University of Connecticut, Storrs, CT 06269, USA
* Correspondence: mousa.khalafi@kashanu.ac.ir (M.K.); maria-luz.fernandez@uconn.edu (M.L.F.)

Supplementary Table S1. Search strategy.

Databases	Search strategy	Results
PubMed	(("time-restricted feeding"[Title/Abstract] OR "time-restricted feeding"[Title/Abstract] OR "time-restricted eating"[Title/Abstract] OR "time-restricted eating"[Title/Abstract] OR "time-restricted diet"[Title/Abstract] OR "time-restricted diet"[Title/Abstract] OR "time-restricted fasting"[Title/Abstract] OR "time-restricted fasting"[Title/Abstract] OR "intermittent fasting"[Title/Abstract] OR "intermittent energy restriction"[Title/Abstract] OR "alternate fasting"[Title/Abstract] OR "periodic fasting"[Title/Abstract] OR "reduced meal frequency"[Title/Abstract] OR "alternate-day fasting"[Title/Abstract] OR "alternate-day fasting"[Title/Abstract]) AND ("Inflammation"[All Fields] OR "inflammatory"[All Fields] OR "cytokine"[All Fields] OR "adipokine"[All Fields] OR "adipocytokine"[All Fields] OR "interleukin"[All Fields] OR "interleukin-6"[All Fields] OR "interleukin-6"[All Fields] OR "IL-6"[All Fields] OR "IL6"[All Fields] OR "interleukin-10"[All Fields] OR "interleukin-10"[All Fields] OR "il 10"[All Fields] OR "il 10"[All Fields] OR "tumor necrosis factor alpha"[All Fields] OR "tnf alpha"[All Fields] OR "tnf alpha"[All Fields] OR "TNF"[All Fields] OR "C-Reactive protein"[All Fields] OR "Reactive protein"[All Fields] OR "hsCRP"[All Fields] OR "CRP"[All Fields] OR "leptin"[All Fields] OR "adiponectin"[All Fields])) AND ((humans[Filter]) AND (english[Filter]))	263
Web of Science	(TITLE-ABS-KEY ("time-restricted feeding" OR "time restricted feeding" OR "time-restricted eating" OR "time restricted eating" OR "time-restricted diet" OR "time restricted diet" OR "time-restricted fasting" OR "time restricted fasting" OR "intermittent fasting" OR "intermittent energy restriction" OR "alternate fasting" OR "periodic fasting" OR "reduced meal frequency" OR "alternate-day fasting" OR "alternate day fasting") AND TITLE-ABS-KEY ("Inflammation" OR "inflammatory" OR "cytokine" OR "adipokine" OR "adipocyto kine" OR "interleukin" OR "interleukin-6" OR "interleukin 6" OR "IL-6" OR "IL6" OR "interleukin-10" OR "interleukin 10" OR "IL-10" OR "IL 10" OR "tumor necrosis factor alpha" OR "TNF-α" OR "TNF α" OR "TNF" OR "C-Reactive protein" OR "Reactive protein" OR "hsCRP" OR "CRP" OR "leptin" OR "adiponectin"))	460
Scopus	(TS=("time-restricted feeding" OR "time restricted feeding" OR "time-restricted eating" OR "time restricted eating" OR "time-restricted diet" OR "time restricted diet" OR "time-restricted fasting" OR "time restricted fasting" OR "intermittent fasting" OR "intermittent energy restriction" OR "alternate fasting" OR "periodic fasting" OR "reduced meal frequency" OR "alternate-day fasting" OR "alternate day fasting")) AND TS=("Inflammation" OR "inflammatory" OR "cytokine" OR "adipokine" OR "adipocytokine" OR "interleukin" OR "interleukin-6" OR "interleukin 6" OR "IL-6" OR "IL6" OR "interleukin-10" OR "interleukin 10" OR "IL-10" OR "IL 10" OR "tumor necrosis factor alpha" OR "TNF-α" OR "TNF α" OR "TNF" OR "C-Reactive protein" OR "Reactive protein" OR "hsCRP" OR "CRP" OR "leptin" or "adiponectin")	413

Supplementary Table S2. Risk of bias assessment.

Authors & Year	Criteria 1	Criteria 2	Criteria 3	Criteria 4	Criteria 5	Criteria 6	Criteria 7	Criteria 8	Criteria 9	Criteria 10	Criteria 11	Total
Bhutan et al. 2013 [31,32]	✓	✓	✓	✓	x	x	x	x	✓	✓	✓	7
Cho et al. 2019 [33]	✓	✓	✓	✓	x	x	✓	x	x	✓	✓	7
Cienfuegos et al. 2020 [34]	✓	✓	x	✓	x	x	x	x	✓	✓	✓	6
Gabel et al. 2019 [35]	✓	✓	x	✓	x	x	x	x	x	✓	✓	5
Haganes et al. 2022 [36]	✓	✓	x	✓	x	x	x	✓	✓	✓	✓	7
Kord Varkaneh et al. 2022 [37]	✓	✓	✓	✓	x	x	x	x	x	✓	✓	6
Kord Varkaneh et al. 2023 [38]	✓	✓	x	✓	✓	✓	x	✓	x	✓	✓	8
Kotarsky et al. 2021 [39]	✓	✓	x	✓	x	x	x	✓	x	✓	✓	6
Lao et al. 2023 [40]	✓	x	x	✓	x	x	x	✓	x	✓	✓	5
Manoogian et al. 2022 [41]	✓	✓	✓	✓	x	x	x	✓	x	✓	✓	7
Martens et al. 2020 [42]	✓	✓	x	✓	x	x	✓	✓	x	✓	✓	7
Miranda et al. 2018 [43]	✓	✓	x	✓	x	x	x	✓	x	✓	✓	6
Moro et al. 2016 [44]	✓	✓	?	✓	x	x	✓	✓	x	✓	✓	7
Moro et al. 2020 [45]	✓	✓	?	✓	x	x	✓	✓	x	✓	✓	7
Moro et al. 2021 [46]	✓	✓	?	✓	x	✓	✓	✓	x	✓	✓	8
Schroder et al. 2021 [47]	✓	x	x	✓	x	x	x	x	x	✓	✓	4
Stratton et al. 2020 [48]	✓	✓	x	✓	x	x	x	✓	x	✓	✓	6
Sutton et al. 2018 [49]	✓	✓	✓	✓	x	x	x	✓	x	✓	✓	7
Varady et al. 2013 [50]	✓	✓	x	x	x	x	x	✓	x	✓	✓	5
Xie et al. 2022 [51]	✓	✓	x	✓	x	?	✓	✓	x	✓	✓	7
Zhang et al. 2022 [52]	✓	✓	x	✓	x	x	x	✓	x	✓	✓	6

(1) Eligibility Criteria specified, (2) Random allocation of participants, (3) Allocation concealed, (4) Groups similar at baseline, (5) Subjects blinded, (6) Therapists blinded, (7) Assessors blinded, (8) Outcome measures assessed in 85% of participants, (9) Intention to treat analysis, (10) Reporting of between group statistical comparison, (11) Point measures and measures of variability reported for main effects. low (✓), high (x) and unclear (?)

Model	Study name	Std diff in means	Standard error	Variance	Lower limit	Upper limit	Z-Value	p-Value
	Cienfuegos et al.2020	0.145	0.317	0.100	-0.476	0.765	0.458	0.647
	Gabel et al. 2019	-0.251	0.398	0.159	-1.032	0.530	-0.629	0.529
	Lao et al. 2023	-0.152	0.386	0.149	-0.908	0.604	-0.394	0.694
	Martens et al. 2020	0.082	0.333	0.111	-0.572	0.735	0.245	0.806
	Miranda et al. 2018	0.240	0.310	0.096	-0.368	0.848	0.774	0.439
	Moro et al. 2016	-0.669	0.352	0.124	-1.360	0.022	-1.899	0.058
	Moro et al. 2020	-1.127	0.538	0.290	-2.182	-0.072	-2.095	0.036
	Moro et al. 2021	-0.901	0.469	0.220	-1.821	0.019	-1.919	0.055
	Sutton et al. 2018	0.600	0.511	0.261	-0.402	1.601	1.173	0.241
	Zhang et al. 2022	0.110	0.278	0.077	-0.434	0.654	0.396	0.692
Random		-0.131	0.146	0.021	-0.417	0.156	-0.893	0.372

Statistics for each study

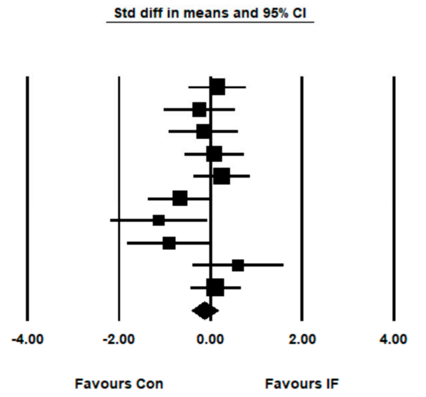

Supplementary Figure S1. Forest plot of the effects of IF training versus CON on IL-6. Data are reported as SMD (95% confidence limits). SMD: standardized mean difference.

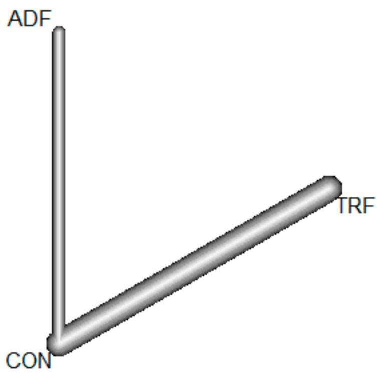

Supplementary Figure S2. Network geometric map of studies investigating the effect of IF training on IL-6.

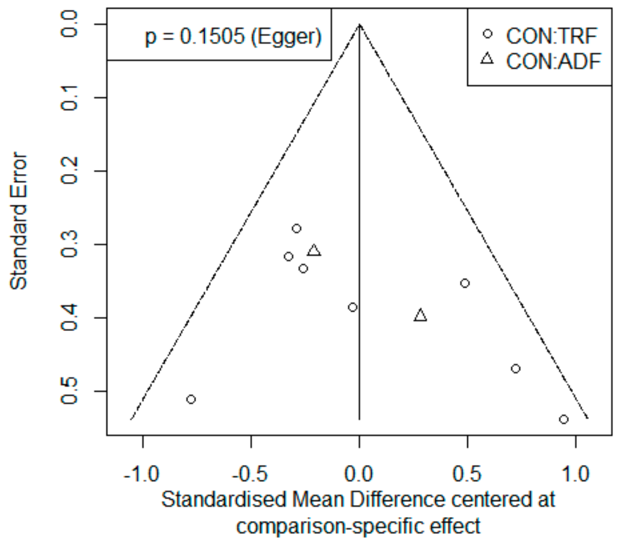

Supplementary Figure S3. Network meta-analysis of funnel plots for IL-6.

Model	Study name	Std diff in means	Standard error	Variance	Lower limit	Upper limit	Z-Value	p-Value
	Cienfuegos et al.2020	-0.114	0.316	0.100	-0.734	0.507	-0.359	0.720
	Gabel et al. 2019	-0.203	0.398	0.158	-0.983	0.577	-0.510	0.610
	Lao et al. 2023	-0.466	0.390	0.152	-1.231	0.299	-1.194	0.232
	Miranda et al. 2018	0.349	0.311	0.097	-0.262	0.959	1.120	0.263
	Moro et al. 2016	-0.767	0.355	0.126	-1.463	-0.070	-2.158	0.031
	Moro et al. 2020	0.015	0.500	0.250	-0.965	0.995	0.031	0.976
	Moro et al. 2021	-0.752	0.463	0.214	-1.659	0.155	-1.625	0.104
	Zhang et al. 2022	-0.342	0.279	0.078	-0.890	0.205	-1.226	0.220
	Xie et al. 2022	-0.546	0.237	0.056	-1.010	-0.082	-2.306	0.021
Random		-0.315	0.120	0.014	-0.551	-0.079	-2.615	0.009

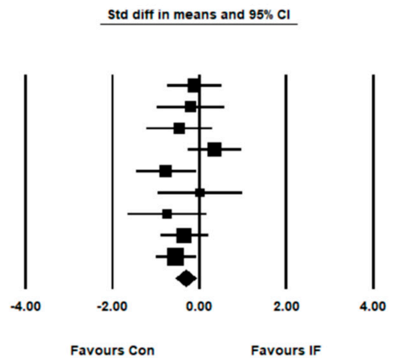

Supplementary Figure S4. Forest plot of the effects of IF training versus CON on TNF-α. Data are reported as SMD (95% confidence limits). SMD: standardized mean difference.

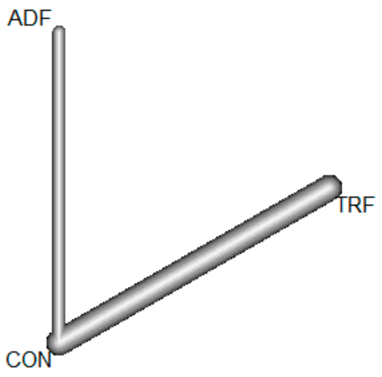

Supplementary Figure S5. Network geometric map of studies investigating the effect of IF training on TNF-α.

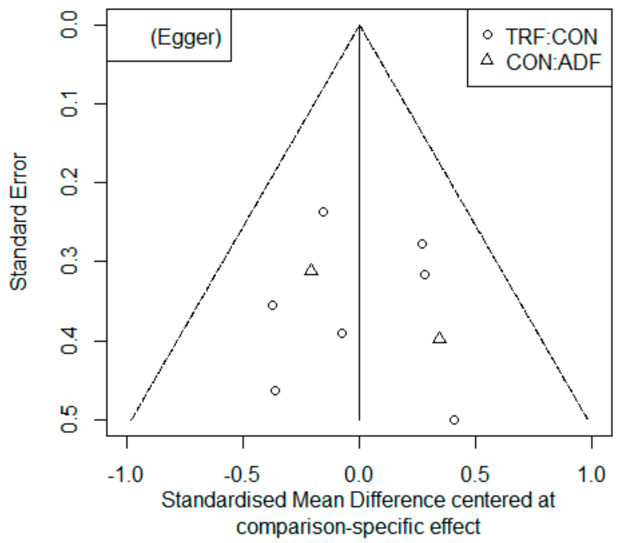

Supplementary Figure S6. Network meta-analysis of funnel plots for TNF-α.

Model	Study name	Std diff in means	Standard error	Variance	Lower limit	Upper limit	Z-Value	p-Value
	Bhutani et al. 2013	0.000	0.354	0.125	-0.693	0.693	0.000	1.000
	Cho et al. 2019	-0.902	0.597	0.356	-2.072	0.268	-1.512	0.131
	Gabel et al. 2019	0.000	0.397	0.158	-0.778	0.778	0.000	1.000
	Kord Varkaneh et al. 2022	-0.645	0.310	0.096	-1.252	-0.038	-2.083	0.037
	Kord Varkaneh et al. 2023	-1.094	0.320	0.102	-1.721	-0.467	-3.421	0.001
	Kotarsky et al. 2021	-0.371	0.441	0.194	-1.235	0.493	-0.842	0.400
	Lao et al. 2023	-0.237	0.387	0.149	-0.995	0.520	-0.614	0.539
	Manoogian et al. 2022	-0.015	0.172	0.030	-0.352	0.323	-0.086	0.932
	Martens et al. 2020	-0.137	0.343	0.118	-0.810	0.536	-0.398	0.690
	Schroder et al. 2021	0.000	0.365	0.133	-0.716	0.716	0.000	1.000
	Sutton et al. 2018	-0.099	0.500	0.250	-1.079	0.882	-0.197	0.844
	Varady et al. 2013	-0.258	0.367	0.134	-0.977	0.460	-0.704	0.481
	Xie et al. 2022	0.013	0.233	0.054	-0.444	0.469	0.054	0.957
	Zhang et al. 2022	0.275	0.322	0.104	-0.356	0.906	0.854	0.393
Random		-0.198	0.100	0.010	-0.394	-0.002	-1.981	0.048

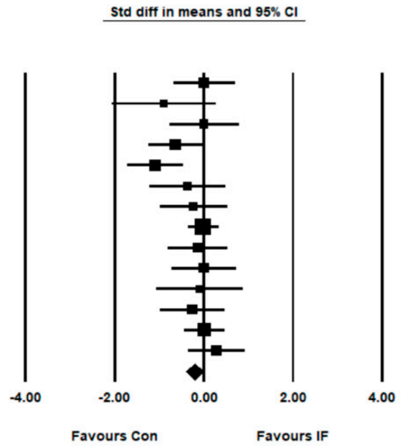

Supplementary Figure S7. Forest plot of the effects of IF training versus CON on CRP. Data are reported as SMD (95% confidence limits). SMD: standardized mean difference.

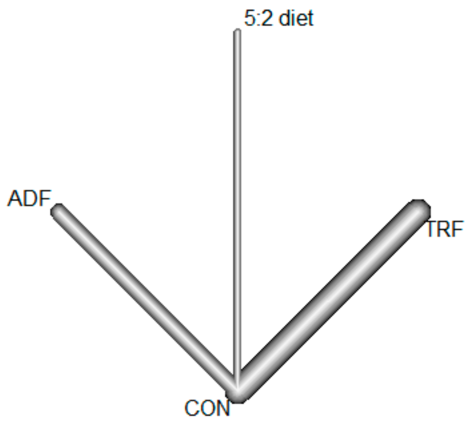

Supplementary Figure S8. Network geometric map of studies investigating the effect of IF training on CRP.

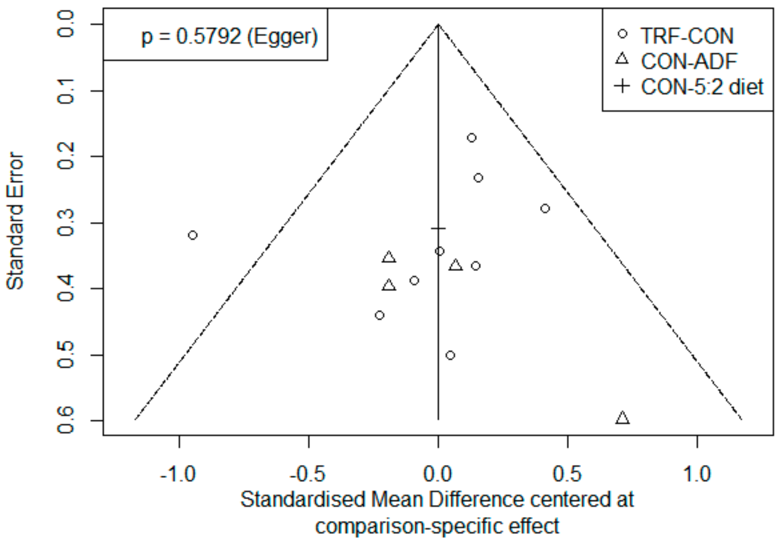

Supplementary Figure S9. Network meta-analysis of funnel plots for CRP.

Model	Study name	Std diff in means	Standard error	Variance	Lower limit	Upper limit	Z-Value	p-Value
		Statistics for each study						
	Bhutani et al. 2013	-0.391	0.357	0.127	-1.091	0.308	-1.096	0.273
	Haganes et al. 2022	-0.160	0.283	0.080	-0.715	0.395	-0.566	0.572
	Miranda et al. 2018	-0.277	0.310	0.096	-0.885	0.332	-0.891	0.373
	Moro et al. 2016	-0.404	0.346	0.120	-1.083	0.275	-1.166	0.244
	Moro et al. 2021	-1.674	0.520	0.270	-2.692	-0.655	-3.221	0.001
	Stratton et al. 2020	0.038	0.392	0.154	-0.731	0.806	0.096	0.924
	Varady et al. 2013	-0.516	0.371	0.138	-1.244	0.211	-1.391	0.164
	Zhang et al. 2022	-1.476	0.309	0.095	-2.081	-0.872	-4.785	0.000
Random		-0.572	0.205	0.042	-0.973	-0.171	-2.794	0.005

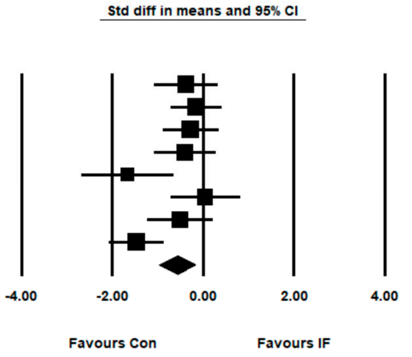

Supplementary Figure S10. Forest plot of the effects of IF training versus CON on leptin. Data are reported as SMD (95% confidence limits). SMD: standardized mean difference.

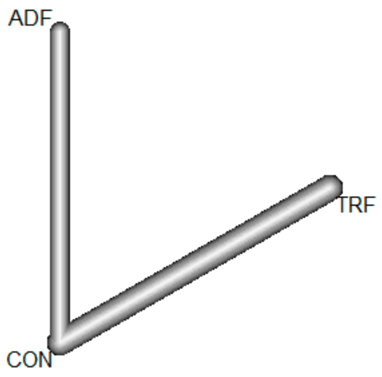

Supplementary Figure S11. Network geometric map of studies investigating the effect of IF training on leptin

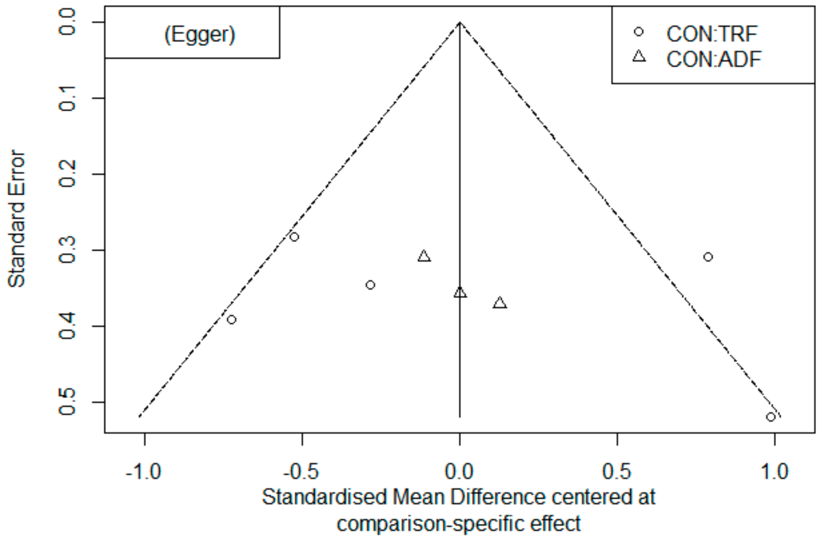

Supplementary Figure S12. Network meta-analysis of funnel plots for leptin.

Model	Study name	Std diff in means	Standard error	Variance	Lower limit	Upper limit	Z-Value	p-Value
	Bhutani et al. 2013	-0.701	0.364	0.133	-1.415	0.013	-1.925	0.054
	Haganes et al. 2022	-0.225	0.284	0.081	-0.782	0.331	-0.794	0.427
	Miranda et al. 2018	-0.286	0.311	0.096	-0.895	0.322	-0.923	0.356
	Moro et al. 2016	0.440	0.347	0.120	-0.241	1.120	1.267	0.205
	Moro et al. 2020	0.330	0.503	0.253	-0.656	1.317	0.656	0.512
	Moro et al. 2021	1.565	0.511	0.261	0.563	2.567	3.062	0.002
	Stratton et al. 2020	-0.049	0.392	0.154	-0.818	0.719	-0.126	0.900
	Varady et al. 2013	0.411	0.369	0.136	-0.312	1.135	1.115	0.265
	Zhang et al. 2022	-0.495	0.281	0.079	-1.046	0.057	-1.759	0.079
Random		0.035	0.193	0.037	-0.344	0.414	0.180	0.857

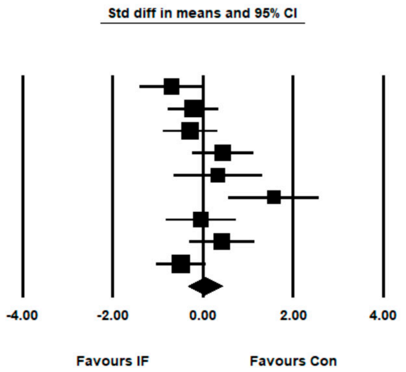

Supplementary Figure S13. Forest plot of the effects of IF training versus CON on adiponectin. Data are reported as SMD (95% confidence limits). SMD: standardized mean difference.

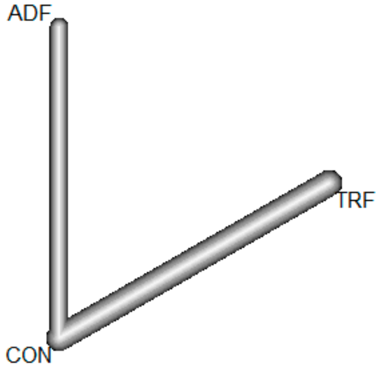

Supplementary Figure S14. Network geometric map of studies investigating the effect of IF training on adiponectin.

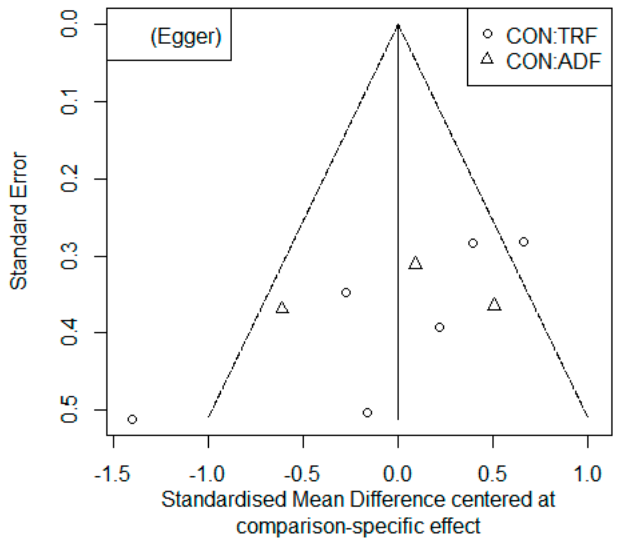

Supplementary Figure S15. Network meta-analysis of funnel plots for adiponectin.
